# Study protocol for a randomized controlled multinational trial to analyze the effects of an app-based patient education textbook (Ready4OP App) to increase health literacy before major oncologic surgery and its potential effects on surgical outcomes in Slovenia, Spain, and Germany—the APERIO Trial

**DOI:** 10.1186/s13063-026-10052-7

**Published:** 2026-11-05

**Authors:** Svenja Sliwinski, Franziska J. Stickl, S. Fatima Faqar-Uz-Zaman, Charlotte Detemble, Dora Zmuc, Jan Zmuc, Gasper Pilko, Cristina Dopazo, Maria Martinez-Lopez, Armin Wiegering, Andreas A. Schnitzbauer

**Affiliations:** 1 Department for General, Visceral, Transplant and Thoracic Surgery, Frankfurt University Hospital, Goethe University Frankfurt/Main, Theodor-Stern-Kai 7, Frankfurt Am Main, 60590 Germany; 2 Klinik Für Chirurgie, Ruhr Universität Bochum, Universitätsklinikum Knappschaftskrankenhaus Bochum, In Der Schornau 23-25, Bochum, 44892 Germany; 3 MCL Medical Center Ljubljana, Ljubljana, Slovenia; 4 Department of Surgical Oncology, Institute of Oncology Ljubljana, Ljubljana, Slovenia; 5 Department of Hepatic and Biliary Surgery and Transplants, Vall d’Hebron Barcelona Hospital Campus, Barcelona, 08035 Spain

**Keywords:** Health literacy, Prehabilitation, Major surgery, Perioperative management, Digitalization, App

## Abstract

**Background:**

Low levels of health literacy (HL) are associated with worse health outcomes and increased mortality. Surgery is among the top three risks of dying in a hospital worldwide. An improvement in health literacy is known to be associated with better healthcare outcomes. To date, there is no data available, on whether a learning instrument can improve health literacy in surgical oncologic patients or if it influences short-term surgical outcomes.

**Methods:**

The APERIO trial is a multicenter prospective randomized controlled trial comparing a digital app-based preparation platform with the hospitals’ standard care. The trial seeks to enroll 382 patients in three different countries who will be randomized in either the intervention or the control group. The primary objective is to evaluate the efficacy of the Ready4OP app in improving health literacy in surgical oncologic patients. The secondary endpoints are investigating 90-day mortality rates, compliance, and adherence to Ready4OP as a digital learning platform, to increase equality of cancer patients’ health literacy in three different European countries and to evaluate the influence of an app-based educational platform on surgical oncologic short-term outcomes and morbidity.

**Discussion:**

We form the hypothesis that providing a digital app-based preparation platform for patients before major surgical oncologic procedures will reduce the number of badly informed patients from 60 to 45% as measured by standardized questionnaires. Therefore, the APERIO trial aims to evaluate the efficacy of the Ready4OP app in improving health literacy in surgical oncologic patients.

**Trial registration:**

Trial registered in the German Clinical Trial Registry (DRKS00031507, Registered on May 22 2023), Goethe University Ethics approval 09.05.2023 (2023-1173).

**Supplementary Information:**

The online version contains supplementary material available at https://doi.org/10.1186/s13063-026-10052-7.

## Administrative information

Note: the numbers in curly brackets in this protocol refer to Standard Protocol Items: Recommendations for Interventional Trials (SPIRIT) checklist item numbers. The order of the items has been modified to group similar items (see http://www.equator-network.org/reporting-guidelines/spirit-2013-statement-defining-standard-protocol-items-for-clinical-trials/).

**Table:** 

Title {1}	A Randomized Controlled Multinational Trial to Analyze the Effects of an App-Based Patient Education Textbook (Ready4OP App) to Increase Health Literacy Before Major Oncologic Surgery and Its Potential Effects on Surgical Outcomes in Slovenia, Spain, and Germany – The APERIO Trial
Trial registration {2a and 2b}.	Trial registered in the German Clinical Trial Registry (DRKS00031507, 22.05.2023), Goethe University Ethics approval 09.05.2023 (2023-1173).
Protocol version {3}	Protocol version number 1.1, March 30th, 2023.
Funding {4}	The study is funded by Goethe University Frankfurt/Main.
Author details {5a}	Svenja Sliwinski^1*^, Franziska J. Stickl^1*^, S. Fatima Faqar-Uz-Zaman^1^, Charlotte Detemble^2^, Dora Zmuc^3^, Jan Zmuc^4^, Gasper Plisko^5^, Cristina Dopazo^5^, Maria Martinez-Lopez^5^, Armin Wiegering^1^, and Andreas A. Schnitzbauer^1,2^ ^1^Department for General, Visceral, Transplant and Thoracic Surgery, Frankfurt University Hospital, Goethe University Frankfurt/Main, Theodor-Stern-Kai 7, 60590, Frankfurt/Main, Germany ^2^Universitätsklinikum Knappschaftskrankenhaus Bochum, Ruhr Universität Bochum, Klinik für Chirurgie, In der Schornau 23–25, 44892 Bochum, Germany ^3^MCL Medical Center Ljubljana, Ljubljana, Slovenia ^4^Department of Surgical Oncology, Institute of Oncology Ljubljana, Ljubljana, Slovenia. ^5^Department of Hepatic and Biliary Surgery and Transplants, Vall d'Hebron Barcelona Hospital Campus, 08035 Barcelona, Spain *Authors contributed equally.
Name and contact information for the trial sponsor {5b}	Prof. Dr. Andreas A. Schnitzbauer Universitätsklinikum Knappschaftskrankenhaus Bochum, Ruhr Universität Bochum, Klinik für Chirurgie, In der Schornau 23–25, 44892 Bochum, Germany andreas.schnitzbauer@ruhr-uni-bochum.de
Role of sponsor {5c}	The sponsor manages the study and feedbacks the reports and the writing of the paper.

## Introduction

### Background and rationale {6a}

Health literacy is crucial for patient outcomes, yet research shows that nearly 60% of the general population struggles with adequate comprehension [1]. The APERIO trial aims to bridge this gap by assessing whether an app-based intervention (Ready4OP) can improve health literacy and patient autonomy before major oncologic surgeries compared to standard care.

Previous studies have demonstrated that low health literacy is associated with increased postoperative complications, longer hospital stays, and higher mortality. However, interventional studies addressing health literacy in surgical patients remain scarce, particularly in the context of digital education tools. Digital health interventions have emerged as promising tools to improve patient education and engagement, with systematic reviews demonstrating their potential to enhance health literacy and patient- reported outcomes, although evidence in surgical settings remains limited and heterogenous [2].

Recent studies evaluating digital perioperative interventions suggest that app-based tools may improve patient engagement, functional recovery, and adherence to prehabilitation programs, but high-quality randomized controlled trials specifically targeting health literacy as a primary outcome are scarce [3]. Furthermore, prior work has highlighted variability in effectiveness, as well as challenges related to usability, accessibility, and implementation across different populations. In addition, preliminary usability and feasibility studies of app-based perioperative interventions have demonstrated acceptable safety profiles and high patient acceptance, supporting further evaluation in randomized controlled settings [4]. However, robust evidence on their direct impact on health literacy and downstream clinical outcomes remains lacking.

The primary objective is to evaluate whether the Ready4OP app reduces the proportion of patients with inadequate or problematic health literacy at the preoperative assessment compared to standard care. Therefore, the APERIO trial addresses a critical gap by evaluating, in a multinational randomized controlled design, whether a structured, app-based educational intervention can meaningfully improve health literacy in patients undergoing major oncologic surgery and potentially influence perioperative outcomes.

Because the intervention is delivered via smartphone application, digital access and basic digital competency are prerequisites for participation. Consequently, patients unable to operate a smartphone independently are not eligible for inclusion. This may introduce selection bias towards patients with greater digital affinity and may limit generalizability of the findings.

### Objectives {7}

#### Primary objective

- We hypothesize that the use of the Ready4OP app will reduce the proportion of patients with inadequate health literacy at the preoperative assessment by 15% compared to standard care.

#### Secondary objectives

- To investigate 90-day mortality rates, compliance and adherence to Ready4OP as a digital learning platform, and its impact on surgical outcomes.
- To increase the equality of cancer patients’ health literacy in three different European Union countries—Slovenia, Germany, and Spain (indicator for improvement measurement: HLS-EU-Q47 score).
- To evaluate the influence of an app-based educational platform on surgical oncologic short-term outcomes and morbidity.
- To analyze adherence and use of a digital learning platform.

#### Hypothesis

The hypothesis is that providing a digital app-based preparation platform for patients before major surgical oncologic procedures will reduce the number of badly informed patients from 60 to 45% as measured by the standardized questionnaires.

### Trial design {8}

**Fig. 1:**
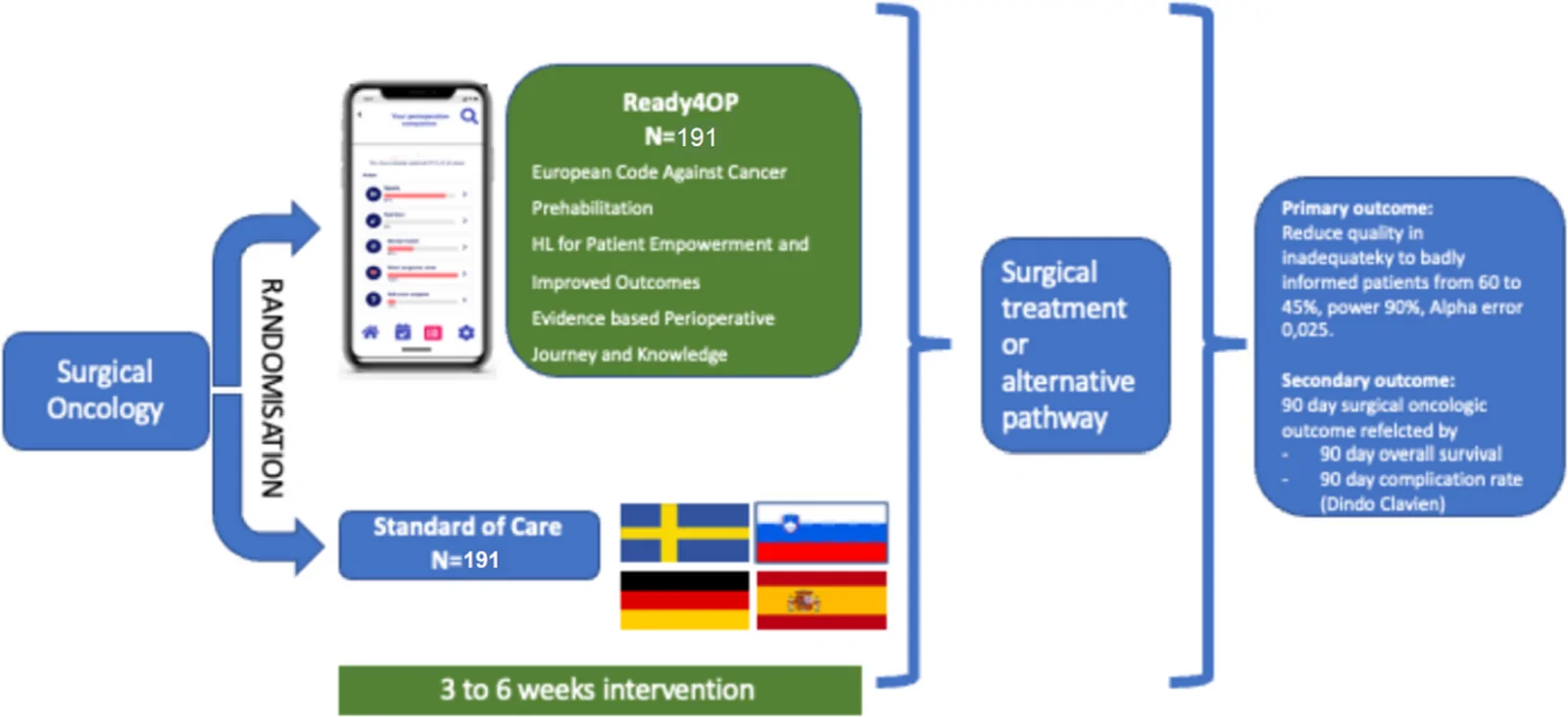
Study design of the APERIO trial

A multicenter, randomized controlled trial involving 382 patients across three countries, conducted between August 2023 and June 2027 (Fig. 1). Patients are randomized per site into intervention (Ready4OP App) and control groups. The patient allocation ratio is 1:1. The framework of the trial is superiority. The randomization reduces selection bias and allocates the patients to the treatment groups. Compliance and use of the app will also be a critical factor that can be included in the subgroup analysis. To minimize variability between participating countries, all centers apply identical eligibility criteria, intervention procedures, investigator training, outcome definitions and electronic case report forms according to the protocol. In addition, adjusted statistical analyses will include country as covariate to account for potential structural differences between healthcare systems.

## Methods: participants, interventions, and outcomes

### Study setting {9}

Patient recruitment is taking place at three trial centers in Germany, Slovenia, and Spain. These are all highly experienced academic hospitals. The list of study sites can be obtained upon request from the corresponding author or under https://drks.de/search/de/trial/DRKS00031507. Reporting was done according to the SPIRIT reporting guidelines [5]. Further, the study at hand was compared and checked with the guideline checklist to improve its quality of reporting (Appendix 2). Although all participating centers are academic hospitals within the European Union and serve comparable tertiary surgical populations, differences in demographic characteristics and baseline health literacy between countries may occur. Baseline characteristics will therefore also be reported by country and the analysis adjusted to account for potential baseline differences where appropriate.

### Eligibility criteria {10}

The eligibility criteria for study centers are hospitals that are experienced in major surgeries.

#### Study population

An improvement in health literacy is known to be associated with better healthcare outcomes. Nonetheless, there is still a tremendous lack of health literacy in the EU, even if there have been long-standing efforts to approach this problem. The World Health Organization even included health literacy as one of the key health promotion pillars in their agenda for sustainable development in 2020 [6]. There are no data available if a learning instrument can improve the health literacy in surgical oncologic patients or if it has an effect on short-term surgical outcomes. We estimated that an improvement of 15% in health literacy would be a meaningful result with clinical impact (see also item 14, page 11). In accordance with Rosner B. *Fundamentals of Biostatistics*, 7th ed. Boston, MA: Brooks/Cole; 2011 [7], we hypothesize that an improvement of patients with a low health literacy level from 60 to 45% can be accomplished with the intervention. Using a power of 80% and an alpha value of 0.05, this accounts for 191 patients in each arm without dropouts and a total of 346 patients with a rough dropout rate of 10%.

The sample size calculation is based on the assumption that 60% of patients receiving standard care will present with inadequate or problematic health literacy at the perioperative assessment. We hypothesize that the Ready4OP app will reduce this proportion to 45%, corresponding to an absolute risk reduction of 15%. This effect size was selected based on previous digital educational interventions demonstrating moderate but clinically meaningful improvements in health literacy and patient engagement and was considered a realistic target for a behavioral intervention in a heterogeneous oncologic surgical population.

Sample size was calculated using a two-sided chi-square test for comparison of proportions with a significance level of *α* = 0.05, a statistical power of 80%, and an allocation ratio of 1:1. Under these assumptions, 173 patients per treatment group are required. To compensate for an anticipated dropout rate of 10%, the final target sample size was increased to 382 patients (191 per group).

#### Inclusion criteria

Inclusion criteria comprise adults (≥ 18 years) (1), willing to participate with written informed consent (Appendix 1) (2), scheduled for major oncologic surgery^1^ (3) with access to a smartphone (Android or iOS) (4). Participants must possess a compatible smartphone, since there is no funding for giving the trial participants a smartphone to use the application if they do not have one; screened patients without smartphone will be screening failures.

In case a patient is scheduled for an oncologic resection and reveals a benign diagnosis after surgery, the patient will stay included in the trial, as the primary suspicion was for a malignant diagnosis. The same applies to patients who are planned for curative resection but are unresectable and are allocated to alternative oncologic pathways thereafter. All patients will be included in the intention-to-treat analysis.

#### Exclusion criteria

Exclusion criteria comprise patients younger than 18 years (1), anamnestically pregnant women (2), patients unable to understand the rationale of the study (3) or unable to interact with the application (4), patients with planned palliative oncologic surgery (5), patients with an Eastern Cooperative Oncology Group (ECOG) ≥ 3 (6), patients with mental impairment leading to inability to cooperate (7), or patients under guardianship or imprisonment (8).

#### Intervention performance

The intervention consists of the application of the digital learning platform Ready4OP. The intervention group will receive a link to download the application from the study team. The patient then will perform the intervention on his own at home by using the application to learn about perioperative care and prehabilitation in nine subsequent subchapters. The Ready4OP application and all study materials, including informed consent documents, are available in German, Spanish, and Slovenian to ensure accessibility across all participating sites.

### Who will take informed consent? {26a}

Patients slated for elective oncologic surgery will undergo eligibility screening conducted by the study team within outpatient departments. Furthermore, all physicians will receive training and orientation regarding the recruitment process to enhance recruitment efficacy. Eligible patients will receive invitations to partake in the study and written informed consent (Appendix 1) will be procured. The principal investigator or designated study staff will obtain informed consent.

### Additional consent provisions for collection and use of participant data and biological specimens {26b}

There is no data use in ancillary studies planned.

## Interventions

### Explanation for the choice of comparators {6b}

The control group will receive standard surgical education without the Ready4OP app. Standard care was chosen as comparator as it reflects current clinical practice, which typically includes unstructured verbal or written patient education. This allows assessment of the added value of structured digital education.

The intervention group will gain access to and be educated with the Ready4OP app. The Ready4OP app provides structured educational content covering perioperative care, patient rights, and prehabilitation strategies in a digital textbook style.

By choosing comparators and accordingly superiority as the framework, the efficacy of the application Ready4OP can be measured best.

### Intervention description {11a}

**Fig. 2:**
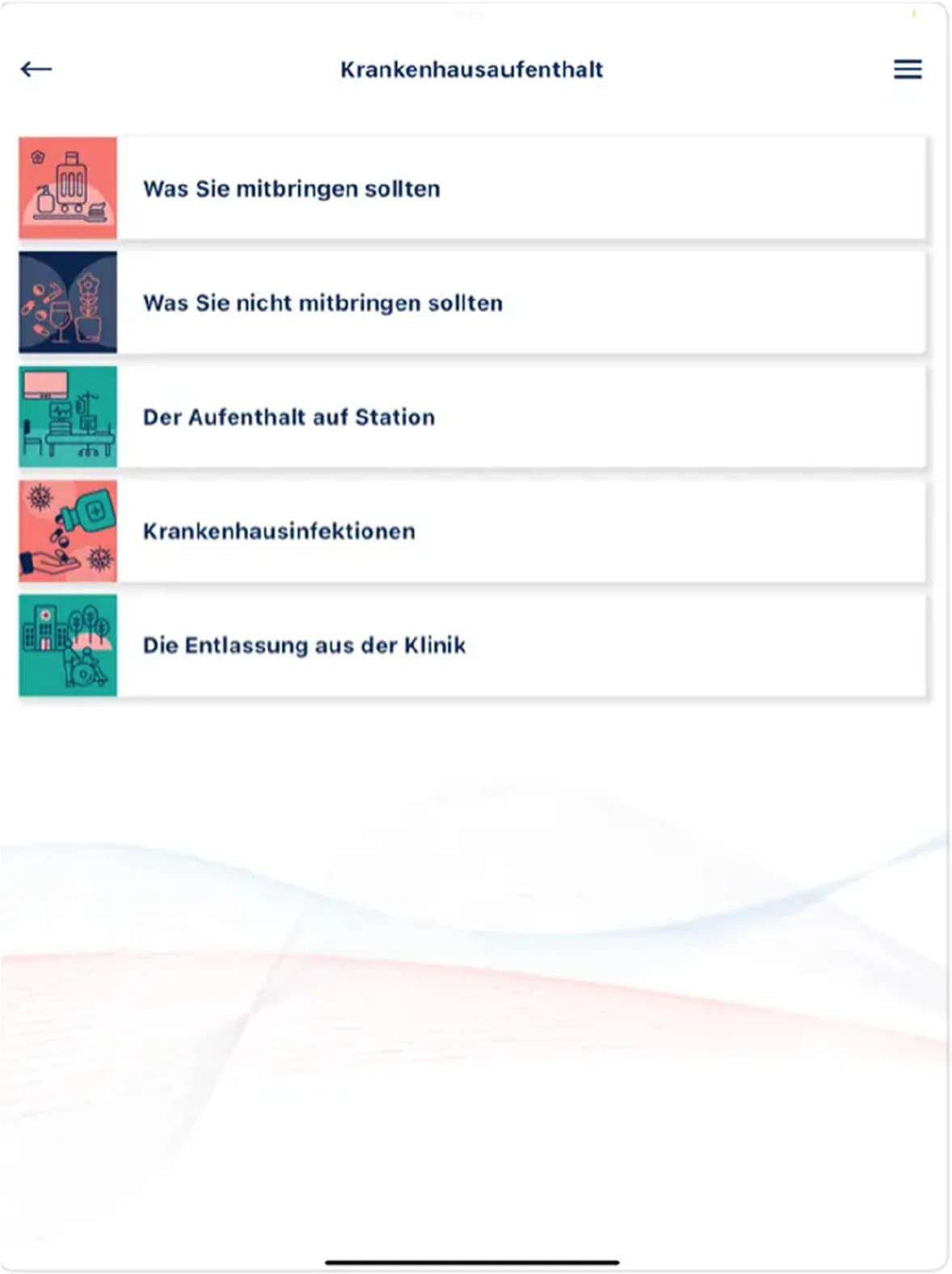
Schematic layout of the app surface Ready4Op

Patients scheduled for major visceral oncologic surgery will be screened for eligibility based on smartphone access (Android/iOS) and surgery timing (1–6 weeks before procedure). If inclusion criteria apply, patients will be included. After informed consent (Appendix 1), eligible patients will be randomized into either the treatment (Ready4OP app) or control (standard care) group. The treatment group will access the app for independent learning on perioperative care and prehabilitation. The Ready4OP app is a patient education tool designed to simplify surgical and perioperative knowledge for adult patients. It includes onboarding, BMI, nutritional habits, fitness level, and surgery date. The app then triggers individualization by featuring nine modules with 52 subcategories covering communication with surgeons, anesthesia, hospital stay, nutrition, exercise, relaxation, postoperative care, and legal aspects (Fig. 2).

The Ready4OP application is a structured perioperative educational platform specifically designed to improve perioperative health literacy, patient empowerment, and communication with healthcare professionals before major oncologic surgery. The intervention consists of 9 educational modules with 52 structured subcategories covering perioperative communication, interpretation of medical reports and medical terminology, informed decision-making, prehabilitation, nutritional, physical activity, risk awareness, perioperative expectations, postoperative recovery, complication awareness, and navigation of healthcare processes. The educational content was developed by an interdisciplinary team of academic surgeons and anesthesiologists and aims to improve patients’ ability to obtain, understand, evaluate and apply perioperative health information. The intervention therefore directly targets core domains assessed by the HLS-EU_Q47 and digital health literacy instruments, including comprehension of medical information, communication with healthcare providers, appraisal of health information, and application of knowledge in health-related decision-making. The application uses algorithm-based individualized navigation pathways that adapt content presentation according to surgical indication, perioperative phase, and user interaction patterns in order to improve usability and patient engagement. At enrolment, participants receive standardized instruction by trained study personnel regarding installation, activation, navigation and use of the Read4OP application. Following this introduction, participants use the application independently throughout the preoperative period. While most hospitals rely on patients’ general knowledge, the app offers broad, evidence-based prehabilitation information without conflicting with standard clinical pathways. Baseline and pre-surgical health literacy will be assessed using three standardized questionnaires. Post-surgery, a 90-day follow-up will track surgical outcomes, complications, and survival. App engagement metrics will assess compliance. No additional study-specific procedures, laboratory tests, or imaging will be required beyond standard clinical care. All demographic data are stored locally on the device; no server data storage or exchange of this information is performed. It aligns with the European Code Against Cancer and WHO recommendations.

### Criteria for discontinuing or modifying allocated interventions {11b}

Participants can withdraw at any time from the study without consequences. 

In addition, the investigator may discontinue a participant from the study at any time if the investigator considers it necessary for any reason including:

- Pregnancy or breastfeeding
- Ineligibility (either arising during the study or retrospective having been overlooked at screening)
- Significant protocol deviation
- Significant non-compliance with the study requirements
- An adverse event which requires discontinuation of the study treatment or results in inability to continue to comply with study procedures
- Disease progression which requires discontinuation of the study treatment or results in inability to continue to comply with study procedures.
- Withdrawal
- Lost to follow-up

The reason for withdrawal will be recorded in the electronic case report forms (eCRF).

### Strategies to improve adherence to interventions {11c}

Engagement data will be tracked, and we conduct the study in a window of opportunity for life changes in patients before surgery, which should lead to high compliance. Furthermore, gamification via the app should encourage app usage.

### Relevant concomitant care permitted or prohibited during the trial {11d}

Patients will continue receiving their standard oncologic treatment. All participants will continue to receive standard perioperative oncological care according to local institutional practice. Standard care includes routine verbal counselling, preoperative consultations and where applicable nutritional counselling. Patients allocated to the control group receive routine preoperative education according to local institutional practice. No structured digital educational intervention is provided fot the control group. Relevant concomitant educational measures such as written educational materials or center-specific perioperative information will be documented throughout the study to allow transparent characterization of the comparator across participating centers.

### Provisions for post-trial care {30}

Standard post-surgical follow-up applies; additional care will be provided for study-related adverse events. 90-day telephone follow-up will be performed. No additional on-site clinical visits are necessary.

Patients will all be treated in accordance with the local standard of care after the trial has been terminated. There is no further requirement to specifically adapt after treatment. All data will be stored in the eCRF. The data collected via the app will be stored in an ISO 27001-certified cloud of a server provider located in Germany. The eCRF data will be stored in the SecuTrial® eCRF system which is a regulatory-cleared provider with servers in Germany. This is a clinical trial with no medical intervention. Patients will receive only evidence-based educational material to measure the effect of this material on their health literacy before major oncologic surgery. Therefore, there will be no increased risk for any participant from any medical intervention.

### Outcomes {12}

Surgery is among the top three risks of dying in a hospital worldwide [8]. The World Health Organization promotes that the reported crude mortality rate after major surgery is 0.5–5%. 25% of patients experience any complication after an in-patient operation [9, 10]. Low levels of health literacy are associated with worse health outcomes and increased mortality [11]. To address this problem, the primary and secondary outcomes of the study are the following:

- Primary endpoint: The primary objective is to evaluate whether the Read4OP app reduces the proportion of patients with inadequate or problematic health literacy at the preoperative assessment compared to standard care, reducing the proportion of patients classified with inadequate/problematic health literacy by an absolute 15% at the pre-surgery assessment.
- Secondary endpoints: 90-day survival, complication rates (Dindo Clavien), app usage and economic impact assessment.

The trial is powered for the primary health literacy endpoint. Analyses of postoperative complications, 90-day survival, readmissions, and other clinical outcomes are exploratory and should therefore be interpreted as hypothesis-generating rather than confirmatory. Primary and secondary outcomes will be investigated after the end of the trial for each patient, which is defined as the date of the last visit 90 days postoperatively or if a patient dies, the last patient contact.

Data will undergo descriptive evaluation and assessment for normal distribution utilizing the Kolmogorov-Smirnov test with Lilliefors correction. Results will be presented as median or mean with standard deviation and interquartile ranges, and/or odds ratios with 95% confidence intervals, where applicable. Survival data will be computed as the time interval from the date of surgery to the date of death, or until censoring if patients are alive at their last visit/contact (90 days follow-up visit).

There will be an intention-to-treat analysis of all patients included in the trial fulfilling the inclusion criteria. Surgical procedures and indications will be categorized based on operation and procedure codes (OPS) and International Statistical Classification of Diseases and Related Health Problems (ICD)−10 codes.

### Participant timeline {13}

Consecutive patients who are planned to undergo major oncologic visceral surgery between August 2023 and June 2027 are considered for study inclusion. The end of enrolment is planned after the inclusion of 382 patients acquired from all centers.

**Fig. 3:**
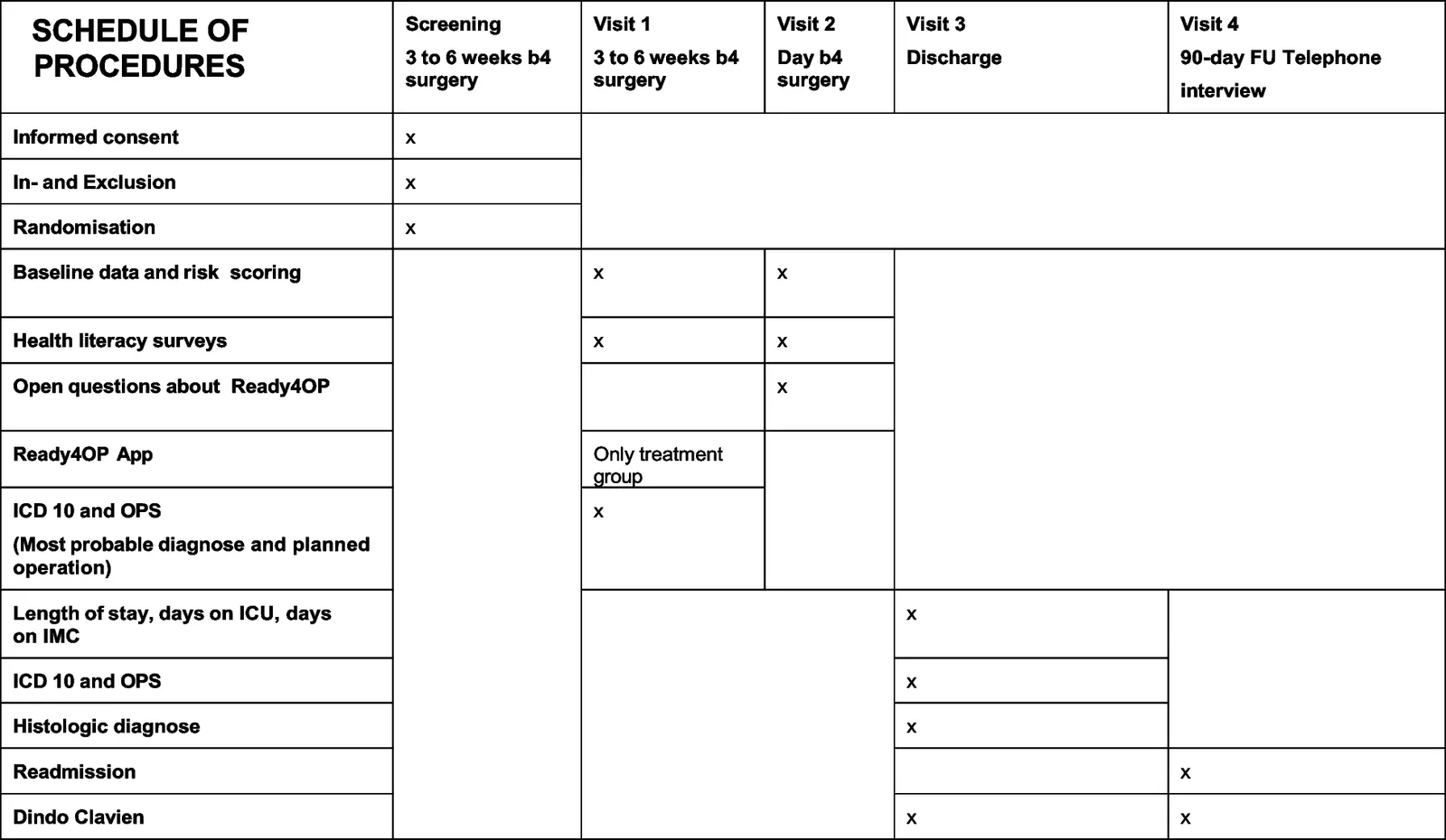
Treatment and visit week scheme. Schedule of enrolment, interventions, and assessments of the APERIO trial

The time schedule of enrolment can be seen in Fig. 3.

Baseline: Informed consent, eligibility assessment, clinical trial site, randomization.

The baseline assessment contains the documentation of the following data:

- Year of birth
- Gender
- ECOG score
- Timed Up and Go testing
- Clinical Risk Analysis Index (RAI-C)-scoring
- 3 HL questionnaires (HL47, HL 11, HL Digi)
- Diagnosis
- Planned Operation (organ system for surgical oncology, dropdown)
- Evaluation of neoadjuvant therapy
- Pre-Surgery: Health literacy assessment (3 HL questionnaires (HL47, HL11, HL Digi)), app usage
- Post-Surgery: Follow-up at discharge and 90 days
- ICD-10 diagnosis
- OPS code of procedure
- Histopathological status
- Date of operation
- Operability (curative/palliative resection, unresectable)
- Postsurgical treatment and duration (intensive care/intermediate care unit, general ward)
- Length of stay
- Dindo Clavien (I–V) (with a structured checklist)
- Planning and receipt of additional therapy, reason for non-application
- Date of follow-up and/or date of death

### Sample size {14}

A total of 382 participants (80% power, 5% alpha error, *n* = 382, 10% drop-out rate) will be included in the study to detect a 15% improvement in health literacy. We assumed that 60% of patients receiving standard care would demonstrate inadequate health literacy at the preoperative assessment. The intervention was hypothesized to reduce this proportion to 45%, corresponding to an absolute reduction of 15%. This effect size was selected based on previous digital educational interventions demonstrating moderate but clinically meaningful improvements in patient education, engagement, and health literacy, together with the association between limited health literacy and adverse perioperative outcomes. Although no validated threshold currently exists and links a specific improvement in health literacy to a defined reduction in surgical complications, a 15% absolute reduction was considered both realistic and clinically meaningful for a behavioral educational intervention.

### Recruitment {15}

Patients will be recruited from participating academic hospitals in their outpatient clinics. All patients planned for major oncologic visceral surgery will be screened for meeting the inclusion criteria. Therefore, reaching an adequate target size is assumed.

## Assignment of interventions: allocation

### Sequence generation {16a}

Computer-generated randomization will allocate participants. Randomization will be performed in SecuTrial®.

After the baseline assessment, the patient will be allocated to the intervention group (use of the Ready4OP app) or the control group (standard treatment at the center). The patient then will receive a Quick Response (QR) code to a specific study version of the Ready4OP app. The study version will be free of charge. The patients in the control arm will be treated according to the site-specific standard.

### Concealment mechanism {16b}

Allocation concealment will be ensured using the centralized randomization module implemented within the SecuTrial® electronic data capture system. The computer-generated allocation sequence is securely stored within the system and remains inaccessible to investigators responsible for participant enrolment until written informed consent has been obtained, eligibility has been confirmed, and all baseline assessments have been completed. Consequently, treatment allocation cannot be predicted or influenced before assignment, thereby minimizing the risk of selection bias. Blinding is not feasible owing to the nature of the intervention.

### Implementation {16c}

Investigators will enroll patients and assign interventions after randomization.

## Assignment of interventions: blinding

### Who will be blinded {17a}

Patients, researchers, and surgeons will not be blinded.

### Procedure for unblinding if needed {17b}

The trial design is open-label; therefore, there is no unblinding procedure.

## Data collection and management

### Plans for assessment and collection of outcomes {18a}

#### Data management and data safety

Authorized representatives from the sponsor, funding source, host institutions, and regulatory authorities will have direct access to trial monitoring, audits, and inspections. Source data, including hospital records, clinical charts, laboratory results, and correspondence, will be documented in the electronic case report form (eCRF). Health literacy questionnaires (HLS-19Q47, HLS19-COM-P-Q11, HLS19-DIGI) will be directly entered into the eCRF as source data. All documents will be securely stored in compliance with EU General Data Protection Regulation (Datenschutz-Grundverordnung, DSGVO), ensuring participant confidentiality by using study codes instead of personal identifiers.

### Plans to promote participant retention and complete follow-up {18b}

The patients will receive extensive information about the study setup and requirements during recruitment. No additional undertakings are required by the patient except for answering questionnaires and in the intervention group usage of the app, which is further assumed to promote patients’ retention. The importance of completion of the follow-up will be stressed. Patients are allowed to stop at any time during the study. Usage of the application is done remotely at the convenience of every patient. No additional outpatient visits are necessary.

### Data management {19}

Data will be managed in compliance with GDPR. All clinical data will be securely stored in the electronic case report form (eCRF) utilizing the SecuTrial® System. (Serious) adverse events ((S)AEs) will be recorded in the eCRF. Backups on the protected research server will be made regularly (once per 3 months). Informed consent and end-of-trial dates will be recorded in the electronic patient dossier and signed paper forms will be stored within our hospital in a locked room. The data systems are validated and have maintenance standard operating procedures (SOPs). The participants will be identified by a study-specific participant number in any database. The name and any other identifying detail will not be included in any study data electronic file.

### Confidentiality {27}

Trial staff will maintain participant anonymity by using only initials and identification (ID) numbers in the CRF and electronic databases. All documents will be securely stored and accessible only to authorized personnel. Data processing will comply with applicable data protection laws and be used solely for scientific purposes, in accordance with Section 24 (1) Hessian Data Protection and Freedom of Information Act (Hessisches Datenschutz- und Informationsfreiheitsgesetz, HDSIG) and Section 12 Hessian Hospital Act (Hessisches Krankenhausgesetz, HKHG).

### Plans for collection, laboratory evaluation and storage of biological specimens for genetic or molecular analysis in this trial/future use {33}

No biological specimens will be stored.

## Statistical methods

### Statistical methods for primary and secondary outcomes {20a}

The study aims to assess whether the Ready4OP app can enhance health literacy and therefore patient understanding and so influence surgical outcomes. A 15% improvement is considered clinically meaningful, reducing the proportion of inadequately informed patients from 60 to 45%. With an 80% power and an alpha of 0.05, the study requires 173 patients per arm, totaling 382 participants, accounting for a 10% dropout rate. Statistical analyses will be undertaken using IBM® SPSS Statistics®. The primary endpoint (proportion of patients with inadequate health literacy) will be compared between groups using the two-sided chi-square test. Effect estimates will be reported as absolute risk differences and odds ratios with 95% confidence intervals. Continuous outcomes (e.g., health literacy scores) will be analyzed using *t*-tests or Mann-Whitney *U* tests depending on distribution. Survival outcomes will be analyzed using Kaplan-Meier methods and log-rank tests. As a supportive analysis, multivariable logistic regression will additionally be performed to adjust for baseline variables including age, sex, country, baseline health literacy, and tumor entity. Results will be reported as adjusted odds ratios with corresponding 95% confidence intervals.

### Interim analyses {21b}

Interim analyses will be conducted solely for safety monitoring purposes. Interim results will not be shared with investigators involved in patient recruitment or outcome assessment. An independent Data Monitoring Committee will review interim safety data and may recommend modification or termination in case of safety concerns. No formal early stopping rules for efficacy are planned due to the low-risk nature of the intervention.

### Methods for additional analyses (e.g., subgroup analyses) {20b}

Subgroup analyses will assess compliance and engagement as well as economic factors.

### Methods in analysis to handle protocol non-adherence and any statistical methods to handle missing data {20c}

Patients in whom data are missing will be treated in accordance with the intention-to-treat principle. Simple imputation will be applied only to missing baseline covariate data which are most likely not going to occur due to the nature of the assessed baseline data. Imputation will not be used for missing primary or secondary outcome values. Missing continuous baseline covariates will be replaced by the median of the observed values for the respective variable, whereas missing categorical baseline covariates will be represented by a separate “missing/unknown” category where appropriate. The extent of missing data for each variable will be reported. The investigators focused on a minimum of data necessary for documentation to keep the overall workload small and to guarantee high compliance on the documentation side. An intention-to-treat analysis will be performed of all patients included in the trial. Subgroup analysis will be performed accordingly with all patients included. No patient will be excluded from analysis as studies on health literacy interventions in surgical oncologic patients are lacking to date and the aim is to gain the greatest increase in knowledge.

### Plans to give access to the full protocol, participant level-data and statistical code {31c}

Not planned.

## Oversight and monitoring

### Composition of the coordinating center and trial steering committee {5d}

The trial is coordinated by Goethe University Frankfurt.

### Composition of the data monitoring committee, its role and reporting structure {21a}

An independent committee will oversee data safety. Direct access will be granted to authorized representatives from the sponsor, the funding source, host institutions, and the regulatory authorities to permit trial-related monitoring, audits, and inspections.

### Adverse event reporting and harms {22}

All adverse events will be recorded and analyzed. This is a clinical trial with no medical intervention. Patients will receive only educational material; there will be no increased risk for any participant from any medical intervention.

### Frequency and plans for auditing trial conduct {23}

Periodic audits will ensure compliance. The process will not be independent from investigators and the sponsor.

### Plans for communicating important protocol amendments to relevant parties (e.g. trial participants, ethical committees) {25}

Any amendments will be submitted to ethics boards and investigators.

### Dissemination plans {31a}

The results of the study are going to be published in a peer-reviewed journal; the data will be anonymized. The datasets used and/or analyzed during the current study can be made available by the corresponding author upon reasonable request. Both positive and negative results will be reported.

## Discussion

Low health literacy is increasingly recognized as an important determinant of perioperative outcomes and healthcare utilization. Patients with limited health literacy are more likely to experience difficulties understanding medical information, adhering to perioperative recommendations, participating in shared decision-making, and navigating complex healthcare systems. Previous studies have demonstrated associations between low health literacy and increased postoperative complications, prolonged hospitalization, reduced treatment adherence, and poorer overall health outcomes [12].

Despite the growing relevance of digital patient education and perioperative optimization strategies, evidence regarding structured digital interventions specifically targeting perioperative health literacy remains limited.

The APERIO trial addresses this gap by evaluating the Ready4OP application in a multinational randomized controlled setting involving surgical oncology patients form Germany, Spain, and Slovenia. To our knowledge, this is among the first multicenter randomized trials specifically designed to investigate whether a structured app-based perioperative educational intervention can improve health literacy before major oncology surgery and potentially influence perioperative outcomes. The multinational design may improve generalizability across different healthcare systems and cultural settings and allows evaluation of the intervention in a heterogeneous real-world surgical population.

A particular strength of this study is the use of validated multidimensional health literacy instruments, including the HLS-EU-Q47 and digital health literacy assessments, which evaluate patients’ abilities to obtain, understand, appraise, and apply health information. These domains directly correspond to the conceptual framework of the Ready4OP intervention, which was specifically designed to improve perioperative communication, understanding of medical information, informed decision-making, and navigation of perioperative care pathways. Digital educational interventions have previously demonstrated moderate but clinically meaningful improvements in health literacy, patient engagement and preventive medicine. However, evidence in surgical populations remains sparse and heterogenous, highlighting the need for adequately designed randomized trials.

The study additionally explores whether improvements in perioperative health literacy may translate into clinically relevant downstream effects, including postoperative complications. Although the trial is not powered primarily for major clinical endpoints, it may provide important exploratory data regarding the relationship between perioperative health literacy and surgical recovery. Such findings could contribute to the development of future perioperative patient-centered educational strategies and digital prehabilitation concepts.

Several limitations should be considered.

First, because the intervention is delivered exclusively via smartphone application, participants must possess compatible mobile devices and sufficient digital competency to use the application independently. Consequently, patients with limited digital access or poor digital literacy are excluded from participation. This may introduce selection bias towards digitally competent individuals and may limit the generalizability of the findings to vulnerable populations with limited access to digital health technologies. To ensure transparency, all screening failures related to digital eligibility will be prospectively documented and reported. However, as digital healthcare delivery becomes increasingly integrated into perioperative medicine, understanding the effectiveness of app-based educational tools in digitally accessible populations remains clinically relevant.

Second, owing to the nature of the educational intervention, participant and investigator blinding is not feasible. Although allocation concealment is maintained until randomization, the open-label design may influence patient-reported outcomes, particularly self-reported health literacy assessed using the HLS-EU-Q47 questionnaire. To minimize this potential source of bias, validated questionnaires are used, and standardized study procedures are implemented across all centers.

Third, the trial is conducted across three academic hospitals in Germany, Slovenia and Spain. Although all participating centers follow an identical study protocol and receive standardized investigator training, routine perioperative education provided as standard care may differ between healthcare systems with respect to verbal counselling, written educational materials, and local clinical practice. To improve transparency, concomitant educational measures will be documented throughout the study, and adjusted analyses will account for study center effects.

Moreover, although the intervention was specifically designed to improve perioperative health literacy, the selected health literacy instruments evaluate broader domains of general and digital health literacy. Consequently, some measured domains may extend beyond the direct perioperative content addressed by the application. However, contemporary concepts of health literacy emphasize transferable competencies related to obtaining, understanding, evaluating, and applying health information across healthcare contexts, which are directly targeted by the intervention.

Finally, the trial is powered for the primary health literacy endpoint. Secondary clinical outcomes, including postoperative complications, 90-day survival, hospital length of stay, and readmission rates, are exploratory and should therefore be interpreted as hypothesis-generating rather than confirmatory.

Despite these limitations, the APERIO trial is, to our knowledge, among the first multinational randomized controlled trials evaluating a structured digital educational intervention specifically designed to improve perioperative health literacy before major oncologic surgery. The multicenter randomized design, standardized intervention, validated health literacy instruments, and comprehensive assessment of clinical and patient-reported outcomes provide a robust methodological framework for evaluating the effectiveness of digital perioperative education. If successful, the Ready4OP application could represent a scalable, resource-efficient strategy to improve perioperative patient education, patient empowerment, and digital health literacy across diverse healthcare systems.

The APERIO trial will provide high-quality evidence regarding the effectiveness of a structured app-based educational intervention for improving perioperative health literacy in patients undergoing major oncologic surgery. The results may contribute to the development of standardized digital patient education strategies and inform future implementation of perioperative digital health interventions in surgical oncology.

### Trial status

Protocol version number 1.1, March 30th, 2023. Recruitment commenced in August 2023 and is expected to end in June 2027.

## Supplementary Information

Supplementary Material 1: Appendix 1. Informed consent materials.

Supplementary Material 2: Appendix 2. SPIRIT Checklist for *Trials.*

## Data Availability

Data access will be restricted to authorized investigators. The datasets used and/or analyzed during the current study will be made available from the corresponding author upon reasonable request in anonymized form. Access to the final dataset will be restricted to authorized investigators who have completed study-specific data protection training, meaning to the principal investigator and the investigators. De-identified datasets may be shared upon reasonable request following approval by the principal investigator and ethics committee only.

## Abbreviations

DSGVO: EU General Data Protection Regulation, Datenschutz-Grundverordnung
ECOG: Eastern Cooperative Oncology Group
eCRF: Electronic case report forms
HDSIG: Hessian Data Protection and Freedom of Information Act, Hessisches Datenschutz- und Informationsfreiheitsgesetz
HKHG: Hessian Hospital Act, Hessisches Krankenhausgesetz
HL: Health literacy
HLS: Health literacy survey
HIPEC: Hyperthermic intraperitoneal chemotherapy
ICD: International Statistical Classification of Diseases and Related Health Problems
ID: Identification
KPI: Key performance indicator
OPS: Operation and procedure codes
QR: Quick Response
RAI-C: Clinical Risk Analysis Index
(S)AE: (Serious) adverse event
SOP: Standard operating procedure
SPIRIT: Standard Protocol Items: Recommendations for Interventional Trials
WHO: World Health Organization

## Footnotes

1 Major oncologic surgery is defined as an elective resection of gastrointestinal, hepatobiliary, pancreatic and other solid organ malignancies requiring general anesthesia and inpatient postoperative care. For example, esophageal cancer resections, gastric cancer resections, pancreatic cancer resections, liver and biliary tract cancer resections, small bowel, colon and rectum resections for cancer, adrenal gland resections for cancer, resection of liver metastases, hyperthermic intraperitoneal chemotherapy (HIPEC) patients.

## References

[1] Schaeffer D, Klinger J, Berens EM, Gille S, Griese L, Vogt D, et al. Health literacy in Germany before and during the COVID-19 pandemic. Gesundheitswesen. 2021;83(10):781–8. 10.1055/a-1560-2479. PMC11248690 34547817

[2] Mukhtar T, Babur MN, Abbas R, Irshad A, Kiran Q. Digital health literacy: a systematic review of interventions and their influence on healthcare access and sustainable development Goal-3 (SDG-3). Pak J Med Sci. 2025;41(3):910–8. 10.12669/pjms.41.3.10639. PMC11911735 40103887

[3] Luo Z, Zhou R, Wei J, Nong K, Peng X, Chen L, et al. Digital health interventions for perioperative patient-reported outcomes: a network meta-analysis. NPJ Digit Med. 2026;9(1). 10.1038/s41746-026-02398-8. PubMed PMID: 41634301. PMC12976316 41634301

[4] Schnitzbauer AA, Detemble C, Faqar-Uz-Zaman SF, Dreilich J, Mohr L, Sliwinski S, et al. Usability testing of a digitized interventional prehabilitation tool for health care professionals and patients before major surgeries: formative and summative evaluation. JMIR Form Res. 2024;8:e59513. 10.2196/59513. PMC11669884 39661439

[5] Chan AW, Tetzlaff JM, Altman DG, Laupacis A, Gøtzsche PC, Krleža-Jerić K, et al. SPIRIT 2013 statement: defining standard protocol items for clinical trials. Ann Intern Med. 2013;158(3):200–7. 10.7326/0003-4819-158-3-201302050-00583. PMC5114123 23295957

[6] Workshop K. Pillars of health promotion and the role of health literacy in the life course approach. Eur J Public Health. 2022;32(Supplement_3):2022. 10.1093/eurpub/ckac129.511.

[7] Kittelson JM. A review of: “Fundamentals of Biostatistics, 7th ed., by B. Rosne.” J Biopharm Stat. 2011;21(5):1046–8. 10.1080/10543406.2011.592364.

[8] Nepogodiev D, Martin J, Biccard B, Makupe A, Bhangu A, Nepogodiev D, et al. Global burden of postoperative death. Lancet. 2019;393(10170):401. 10.1016/S0140-6736(18)33139-8. 30722955

[9] WHO Guidelines for Safe Surgery 2009. Safe Surgery Saves Lives. Geneva: World Health Organization; 2009. PMID: 23762968. 23762968

[10] Nishiwaki K, Ichikawa T. WHO surgical safety checklist and guideline for safe surgery 2009. Masui. 2014;63(3):246–54 PubMed PMID: 24724433. 24724433

[11] Stormacq C, Wosinski J, Boillat E, Van den Broucke S. Effects of health literacy interventions on health-related outcomes in socioeconomically disadvantaged adults living in the community: a systematic review. JBI Evid Synth. 2020;18(7):1389–469. 10.11124/JBISRIR-D-18-00023. 32813388

[12] Dong Q, Liu T, Liu R, Yang H, Liu C. Effectiveness of digital health literacy interventions in older adults: single-arm meta-analysis. J Med Internet Res. 2023;25:e48166. 10.2196/48166. PMC10365623 37379077

