# Supplementary material for: Study protocol for a randomized controlled multinational trial to analyze the effects of an app-based patient education textbook (Ready4OP App) to increase health literacy before major oncologic surgery and its potential effects on surgical outcomes in Slovenia, Spain, and Germany—the APERIO Trial
Source: Trials. 2026 Nov 5;27:650. doi: 10.1186/s13063-026-10052-7 (PMC13737784; doi:10.1186/s13063-026-10052-7)
Supplement: Supplementary file 1 [file 13063_2026_10052_MOESM1_ESM.pdf]

Patienteninformation

APERIO Trial

Eine randomisiert-kontrollierte multinationale Studie zur Analyse einer App-basierten Patientenedukation mittels der Ready4OP App (Patientenlehrbuch) zur Verbesserung der Gesundheitskompetenz vor großen chirurgischen Eingriffen und seiner potenziellen Effekte auf die chirurgischen Ergebnisse in Schweden, Slowenien, Spanien und Deutschland

Sehr geehrte Patientin, sehr geehrter Patient,

wir möchten Sie bitten, sich einen Moment Zeit zu nehmen, um sich von uns über eine klinische Studie unserer Klinik informieren zu lassen. Diese befasst sich mit der Anwendung der Ready4OP App, die von der Patronus Health GmbH (Ausgründung aus der Goethe Universität) entwickelt wurde. Die mündliche Information zur Studie wird ca. 15 Minuten in Anspruch nehmen.

Bitte lesen Sie diese Informationen sorgfältig durch, damit Sie entscheiden können, ob Sie an der Studie teilnehmen möchten oder nicht. Ihr(e) Studien:ärztin/betreuende Studierende werden mit Ihnen die Details zur Studie besprechen und Ihre Fragen beantworten. Danach werden Sie ausreichend Bedenkzeit erhalten, um über eine Teilnahme zu entscheiden.

Wenn Sie an der Studie teilnehmen möchten, bitten wir Sie, die angehängte Einwilligungserklärung zu unterschreiben, wodurch Sie bestätigen, dass Sie die vorgelegten Informationen vollständig gelesen und der Teilnahme an dieser Studie zugestimmt haben. Mit Ihrer Teilnahme an dieser Studie helfen Sie uns, wissenschaftliche Erkenntnisse zu gewinnen.

Hintergrund und Ziel der Studie

Die Gesundheitskompetenz, also das Wissen über medizinische Informationen ist in Europa bei 50-60% aller Patient:innen mangelhaft bis sehr schlecht. Oftmals sind Sie als Patient:in unsicher, was Sie Ihre Ärzt:in fragen sollen, wie Sie sicher kommunizieren können, und welche Informationen Ihnen eigentlich dabei helfen, besser informiert zu sein. Es ist bekannt, dass ein verbessertes Wissen zur eigenen Erkrankung und Therapie unter Umständen mit einer verbesserten medizinischen Versorgung einhergehen kann.

Sie selbst benötigen eine große Operation im Bauchraum. Hier wissen wir, dass Patient:innen häufig mit der Flut der Informationen überfordert sein können. In der App erhalten Sie Informationen zu 9 Kategorien mit insgesamt 52 Unterkapiteln, die Ihnen wertvolle

Informationen darüber geben, was Sie Ihre Ärzte fragen sollten, was Befunde bedeuten und welche Befunde und Krankheitsinformationen Sie zu einem Termin oder in das Krankenhaus mitbringen sollen. Zudem erhalten Sie wichtige und wissenschaftlich belegte Informationen über Bewegung, Atemtraining und Sport vor einer großen Operation. Weiter über die mentale Vorbereitung, Ernährung und schließlich für den Aufenthalt in der Klinik. Hierzu gehört neben den rechtlichen Dingen, wie der Aufklärung, der Zweitmeinung oder auch der Vorsorgevollmacht, ein Erklärvideo darüber, was während der Narkose passiert und abschließend wie ein normaler oder komplikativer postoperativer Verlauf aussehen kann und was das für Sie bedeutet.

Unser Ziel ist es nun herauszufinden, ob die Inhalte der App (Ready4OP App) dazu beitragen, Ihre Gesundheitskompetenz zu dem Themenkomplex „Große Operation und optimale Vorbereitung" zu verbessern. Wir ersetzen dabei nicht das Informationsmaterial vor der Operation, sondern geben Ihnen zusätzliche Informationen in leichter Sprache an die Hand, die im Klinikalltag häufig zu kurz kommen und im vorhergehenden Absatz erläutert wurden.

Welche Verfahren werden im Rahmen der Studie angewendet und untersucht und wie ist der Ablauf der Studie?

Wenn Sie sich für eine Teilnahme entscheiden, werden Sie zufällig per Losverfahren entweder in die Gruppe, die die App nutzen wird, gelost oder Sie werden im Kontrollarm, ohne die App zu nutzen nach Zentrumsstandard behandelt. Aktuell wissen wir nicht, ob die Inhalte der App Ihr Wissen und die Ergebnisse nach der Operation positiv beeinflussen. Es ist auch möglich, dass Sie durch Ihre Teilnahme an dieser Studie keinen Nutzen für Ihre Gesundheit haben werden. Die Ergebnisse dieser Studie können jedoch dazu beitragen, dass für andere Patienten, die Versorgung verbessert wird.

In jedem Fall messen wir bei Einschluss in die Studie und erneut nach 2 bis 6 Wochen direkt vor der Operation Ihre Gesundheitskompetenz mit insgesamt 3 strukturierten und validierten Fragebögen der Europäischen Gesellschaft für Gesundheitskompetenz (47 Fragen zu generellem Wissen zu medizinischen Informationen & 11 Elemente, wie Sie Ihren Arzt Fragen können sowie 10 Elemente, wie Sie Informationen im Internet finden oder in einer App nutzen können. Das Ausfüllen der 3 Bögen dauert insgesamt bei jedem Termin etwa 20 bis 25 Minuten (Gesamt 2-mal). Aus der Differenz der Werte können wir dann sehen, ob sich Ihre Gesundheitskompetenz verbessert hat, gleichblieb, oder sogar schlechter wurde und ob das einen Einfluss auf den postoperativen Verlauf hat.

Wenn Sie dem App-Arm zugeordnet wurden, werden Sie von uns einen Downloadlink für die App erhalten. Wir werden Daten von Ihnen in einer elektronischen Fallakte speichern. Sie nutzen die App oder bereiten sich, während der 2 bis 6 Wochen vor der Operation so vor, wie es Ihnen das Zentrum empfiehlt. Vor der Operation füllen Sie erneut die Fragebögen aus. Ihre

behandelnden Ärzte erheben Ihre Risikofaktoren vor der Operation, dokumentieren Ihren Krankenverlauf und werden Sie bis 90 Tage nach Operation nachverfolgen und Informationen über den Verlauf zwischen Entlassung und 90 Tage telefonisch von Ihnen einholen.

Befinden Sie sich im Kontrollarm werden die gleichen Daten erhoben, wie bei den Patient:innen, die die App benutzt haben.

Sollten Sie sich zur Teilnahme an der Studie entscheiden, werden Sie insgesamt 4-mal von uns kontaktiert: Vor der Studie im Rahmen des Screenings und zur Erhebung der ersten Daten mit den 3 Fragebögen, anschließend am Tag vor der Operation zur erneuten Erhebung mit den gleichen 3 Fragebögen, am Entlasstag und 90 Tage nach der Operation. Das Ausfüllen der Fragebögen erfolgt im Rahmen der regulären Vorstellungstermine, Sie müssen keinesfalls extra zu einem weiteren Termin kommen. Der Abschlusstermin wird telefonisch durchgeführt.

Wir werden Sie, wenn Sie sich im App-Arm befinden darüber hinaus darum bitten uns zu sagen, ob Sie selber Themengebiete in der App vermissen, die wir dann ggfs. neu mit dazu nehmen, da wir davon überzeugt sind, dass die medizinische Versorgung nur gemeinsam mit den Patient:innen verbessert werden kann. Danach ist die Studie für Sie beendet.

Nutzen und Risiken der Studienteilnahme

Die Studienteilnahme hat keinerlei Einfluss auf ihre Behandlung und die Operation. Es bestehen keine körperlichen Risiken. Sie müssen lediglich Fragebögen ausfüllen, die Ihre Gesundheitskompetenz messen und Sie dürfen bzw. sollen, wenn Sie in die entsprechende Gruppe eingeteilt werden, die Ready4OP App nach Belieben nutzen.

Ein möglicher Vorteil könnte sein, dass Sie nach der Intervention eine erhöhte Gesundheitskompetenz aufweisen, wobei bis dato noch völlig unklar ist, ob dies überhaupt einen Einfluss auf Ihren postoperativen Verlauf hat.

Sie haben durch eine Studienteilnahme grundsätzlich auch keinen sicheren unmittelbaren Nutzen. Sie tragen jedoch entscheidend dazu bei, die Fragestellung wissenschaftlich zu beantworten und helfen künftigen Generationen an Patient:innen.

Wie lange dauert die Studie für mich?

Für Sie als Patienten bestehen die studienspezifischen Anwendungen in der zweimaligen Erhebung von 3 Gesundheitskompetenzfragebögen, einer Befragung hinsichtlich Patient: innen-definierter Elemente für die App und einem Telefoninterview 90 Tage nach der Operation, um operationsspezifische Aspekte abzufragen. Es sind keine Nachfolgetermine im

Rahmen dieser Studie geplant. Ebenso sind keine Blutentnahmen geplant, die studienspezifisch sind.

Wer organisiert die Studie?

Die APERIOS Studie wird von der Klinik für Allgemein-, Viszeral-, Transplantations- und Thoraxchirurgie der Universitätsklinik Frankfurt initiiert und durchgeführt. Verantwortlich für die Studie ist die Goethe-Universität Frankfurt am Main.

Was ist, wenn ich zu einem späteren Zeitpunkt nicht mehr an der Studie teilnehmen möchte?

Sie können Ihre Einwilligung jederzeit ohne Angabe von Gründen und ohne Nachteile für Ihre weitere medizinische Versorgung zurückziehen.

Versicherungsschutz

Bei dieser klinischen Studie, bei der wir Ihnen ein Lehrbuch als App zur Verfügung stellen und Ihre Gesundheitskompetenz mit Fragebögen messen, ist keine Versicherung der Studienteilnehmer erforderlich. Wir weisen Sie ferner darauf hin, dass Sie auf dem Weg von und zur Prüfstelle nicht unfallversichert sind.

Vertraulichkeit, Datenverarbeitung und Datenschutz

Während der Studie werden keine medizinischen Befunde in der App erhoben. Sie müssen allerdings ein paar wenige persönliche Informationen in der App elektronisch angeben, die aber nur auf Ihrem Smartphone gespeichert werden und nicht an einen Server übertragen werden. Die für die Studie wichtigen Daten werden in pseudonymisierter* (=codierter) Form in einer speziellen Studiendatenbank (eCRF, elektronische Case Report Form) gespeichert, ausgewertet, für das Datenmanagement und die Auswertung an das *Institut für Biostatistik und Mathematisches Modelling am Fachbereich Medizin der Goethe Universität, Theodor-Stern Kai 7, 60590 Frankfurt am Main* weitergegeben und ausgewertet. Sobald es nach dem Forschungs- oder Statistikzwecken möglich ist, werden die personenbezogenen Daten anonymisiert*.

Die Patronus Health GmbH hat keinen Zugriff auf die Studiendaten, allerdings liefert das Unternehmen sogenannt Key Performance Indikatoren, die die Häufigkeit des Zugriffs auf die App messen, damit wir objektiv nachvollziehen können, wie oft Sie die App benutzen. Allerdings können wir diese Erhebung nicht für jeden Patienten individuell, sondern nur über die gesamte Kohorte nachvollziehen. Dies geschieht also in anonymisierter aggregierter Form.

**Anonymisierung ist das Verändern personenbezogener Daten in der Weise, dass die betroffene Person nicht mehr oder nur mit einem unverhältnismäßig großen Kosten- oder Zeitaufwand identifiziert werden kann.*

**Pseudonymisierung bedeutet, dass keine Angaben von Namen oder Initialen verwendet werden, sondern nur ein Nummern- und/oder Buchstabencode, evtl. mit Angabe des Geburtsjahres. Die Daten sind gegen unbefugten Zugriff gesichert.*

Die Studienleitung wird alle angemessenen Schritte unternehmen, um den Schutz Ihrer Daten gemäß den Datenschutzstandards der Europäischen Union zu gewährleisten. Die Daten sind gegen unbefugten Zugriff gesichert (siehe oben). Mit Einreichen der Studie bei der zuständigen Ethikkommission wurde ein stringentes Datenschutzfolgekonzept vorgelegt.

Die pseudonymisierten Daten werden ausschließlich zu Zwecken dieser Studie verwendet. Sie haben das Recht dieser weiteren Verwendung zu widersprechen.

Die pseudonymisierten Daten werden nach Studienabschluss für 10 Jahre aufbewahrt.

Darüber hinaus haben Sie das Recht, von Prof. Dr. A. Schnitzbauer sowie den Datenschutzbeauftragten Auskunft über die von Ihnen gespeicherten personenbezogenen Daten zu verlangen. Ebenfalls können Sie die Berichtigung unzutreffender Daten sowie die Löschung der Daten oder Einschränkung deren Verarbeitung verlangen, solange sie nicht anonymisiert wurden.

Der Gesamtverantwortliche für die studienbedingte Erhebung personenbezogener Daten ist:

Prof. Dr. Andreas A. Schnitzbauer
Telefon: +49 69 / 6301-87197
Email: Andreas.Schnitzbauer@kgu.de

Bei Anliegen zur Datenverarbeitung und zur Einhaltung der datenschutzrechtlichen Anforderungen können Sie sich an den Datenschutzbeauftragten der Universitätsklinik Frankfurt am Main wenden.

Postadresse: Datenschutzbeauftragte Universitätsklinikum Frankfurt am Main
Tel.: +49 69 / 6301-7235, Fax: +49 69 / 6301-83779
E-Mail: Datenschutz@kgu.de
Universitätsklinikum Frankfurt am Main,
Theodor-Stern-Kai 7,
60590 Frankfurt am Main

Im Falle einer rechtswidrigen Datenverarbeitung haben Sie das Recht, sich bei folgender Aufsichtsbehörde zu melden:

Der Landesbeauftragte für den
Datenschutz und die Informationsfreiheit Hessen
Postfach 3163, 65021 Wiesbaden, Gustav-Stresemann-Ring
Telefon: +49 611 1408 – 0, Telefax: +49 611 1408 - 900 / 901
E-Mail: poststelle@datenschutz.hessen.de
Internet: https://datenschutz.hessen.de/

Für die Zwecke der Studie ist es nützlich, auch Daten aus Ihrer Krankenakte bei Ihrem Hausarzt einzubeziehen. Wir möchten Sie bitten, einer Kontaktaufnahme mit dem Hausarzt und der Weitergabe Ihrer Daten zum Zweck der Studie zuzustimmen und Ihren Hausarzt insoweit von der Schweigepflicht zu entbinden. Dies betrifft z.B. Vorerkrankungen oder den Nachverfolgungszeitraum, wenn hier spezifische Sie betreffende medizinische Fallstricke auftreten sollten.

Was geschieht mit den Ergebnissen der Studie?

Ihre Daten werden in pseudonymisierter Form erfasst und analysiert, in zusammengefasster Form anonymisiert (ohne dass irgendjemand Rückschlüsse auf einen einzelnen Patienten ziehen kann) publiziert und die Ergebnisse somit für andere Ärzte und zukünftige Patienten nutzbar gemacht.

Haben Sie weitere Fragen?

Wenn Sie Fragen bezüglich des Studienablaufs haben, wenden Sie sich bitte an die Betreuer der Studie. Auch bei etwaigen anderen Fragen können Sie gerne die Sie betreuenden Personen ansprechen.

Für Rückfragen stehen Ihnen folgende Personen zur Verfügung:

Ihr/e aufklärende/r Studienarzt/-ärztin:

(Name)

Studienleiter:

Prof. Dr. med. Andreas A. Schnitzbauer
Klinik für Allgemein- und Viszeralchirurgie
Universitätsklinikum Frankfurt am Main
Theodor-Stern-Kai 7, 60590 Frankfurt am Main

Telefon Studienleiter: 069 6301-87197

Für weitere Informationen sowie für Auskünfte über allgemeine Ergebnisse und den Ausgang der Studie steht Ihnen als Leiter der Studie Herr Prof. Dr. med. Andreas A. Schnitzbauer (E-Mail: Andreas.Schnitzbauer@kgu.de) zur Verfügung.

Wir bedanken uns für Ihre Unterstützung. Eine Ausfertigung des Dokumentes ist für den Verbleib bei Ihnen bestimmt.

UNIVERSITÄTS KLINIKUM FRANKFURT
GOETHE-UNIVERSITÄT

Patienteneinwilligung

APERIO TRIAL

Eine randomisiert-kontrollierte multinationale Studie zur Analyse einer App-basierten Patientenedukation mittels der Ready4OP App (Patientenlehrbuch) zur Verbesserung der Gesundheitskompetenz vor großen chirurgischen Eingriffen und seiner potenziellen Effekte auf die chirurgischen Ergebnisse in Schweden, Slowenien, Spanien und Deutschland

Ich bin heute in einem ausführlichen Aufklärungsgespräch über Art, Umfang und Bedeutung dieser klinischen Studie aufgeklärt worden. Dabei wurden u. a. Studienziel und Studiendauer, studienbedingte Erfordernisse und mögliche Risiken und Unannehmlichkeiten im Rahmen der Studienteilnahme besprochen. Die Patienteninformation zur Studie sowie ein Exemplar der Einwilligungserklärung habe ich erhalten und gelesen. In diesem Zusammenhang bestehende Fragen wurden besprochen und beantwortet.

Folgende Fragen/sonstige Aspekte wurden zusätzlich besprochen:

Mir ist bekannt, dass diese Studie in erster Linie der medizinischen Wissenserweiterung dient. Ich stimme der Teilnahme an der oben genannten Studie freiwillig zu. Ich hatte ausreichend Zeit, mich für oder gegen die Teilnahme an der Studie zu entscheiden. Ich weiß, dass ich meine Einwilligung jederzeit ohne Begründung und ohne Nachteile für meine weitere medizinische Versorgung widerrufen kann.

Datenschutz

Mir ist bekannt, dass bei dieser klinischen Studie personenbezogene Daten, insbesondere medizinische Befunde über mich erhoben, gespeichert und ausgewertet werden sollen. Die Verarbeitung der Daten erfolgt nach gesetzlichen Bestimmungen und setzt gemäß Art. 6 Abs. 1 lit. a und Art 9 der Datenschutz-Grundverordnung folgende Einwilligungserklärung voraus:

Ich wurde darüber aufgeklärt und stimme freiwillig zu, dass meine in der Studie erhobenen Daten, insbesondere Angaben über meine Gesundheit, aufgezeichnet und ausgewertet werden. Soweit erforderlich, dürfen die erhobenen Daten pseudonymisiert (verschlüsselt) und zur wissenschaftlichen Auswertung weitergegeben werden an:

- Prof. Dr. med. Andreas A. Schnitzbauer, Klinik für Allgemein- und Viszeralchirurgie, Universitätsklinikum Frankfurt am Main (Studienleiter)
- Institut für Biostatistik und Mathematisches Modelling
- Dritte erhalten keinen Einblick in personenbezogene Unterlagen. Bei der Veröffentlichung von Ergebnissen der Studie wird mein Name ebenfalls nicht genannt. Die personenbezogenen Daten werden anonymisiert, sobald dies nach dem Forschungszweck und von Gesetz wegen möglich ist.

Außerdem erkläre ich mich damit einverstanden, dass autorisierte und zur Verschwiegenheit verpflichtete Beauftragte des Studienleiters, sogenannte Prüfer und ggfs. Monitore, in meine beim Prüfarzt vorhandenen personenbezogenen Daten, insbesondere meine Gesundheitsdaten, Einsicht nehmen, soweit dies für die Überprüfung der ordnungsgemäßen Durchführung und der Datenqualität der Studie notwendig ist. Für diese Maßnahme entbinde ich den Prüfarzt von der ärztlichen Schweigepflicht.

Ich bin damit einverstanden, dass Gesundheitsdaten von meinem Hausarzt für die Zwecke der Studie erhoben werden. Insoweit entbinde ich meinen Hausarzt von der Schweigepflicht (falls nicht gewünscht, bitte streichen)

Name des Hausarztes

……

Mir ist bekannt, dass diese Einwilligung jederzeit schriftlich oder mündlich ohne Angabe von Gründen widerrufen werden kann, ohne dass mir dadurch Nachteile entstehen. Die Rechtmäßigkeit der bis zum Widerruf erfolgten Datenverarbeitung wird davon nicht berührt. In diesem Fall kann ich entscheiden, ob die von mir erhobenen Daten gelöscht werden sollen oder weiterhin für die Zwecke der Studie verwendet werden dürfen.

Frankfurt, ______________ ______________________________

Name der Patient:in Datum Unterschrift

(in Druckbuchstaben)

Frankfurt, ______________ ______________________________

Name der aufklärenden Studien:ärztin/Studierenden Datum Unterschrift

(in Druckbuchstaben)

Bitte beachten Sie, dass die Patienten selbständig und handschriftlich Ihren Namen und Datum eintragen, sowie unterschreiben, bevor eine Prüfärzt:in oder Studierende selbständig Namen und Datum eintragen und unterschreiben.
